# Multiple Roles of MYC in Integrating Regulatory Networks of Pluripotent Stem Cells

**DOI:** 10.3389/fcell.2017.00007

**Published:** 2017-02-03

**Authors:** Luca Fagnocchi, Alessio Zippo

**Affiliations:** ^1^Department of Epigenetics, Fondazione Istituto Nazionale di Genetica Molecolare “Romeo ed Enrica Invernizzi” (INGM)Milan, Italy; ^2^Division of Pathology, Fondazione IRCCS Ca' Granda, Ospedale Maggiore PoliclinicoMilan, Italy

**Keywords:** MYC, pluripotent stem cells, transcription regulatory networks, epigenetics, signaling pathways, epigenetic memory, cancer

## Abstract

Pluripotent stem cells (PSCs) are defined by their self-renewal potential, which permits their unlimited propagation, and their pluripotency, being able to generate cell of the three embryonic lineages. These properties render PSCs a valuable tool for both basic and medical research. To induce and stabilize the pluripotent state, complex circuitries involving signaling pathways, transcription regulators and epigenetic mechanisms converge on a core transcriptional regulatory network of PSCs, thus determining their cell identity. Among the transcription factors, MYC represents a central hub, which modulates and integrates multiple mechanisms involved both in the maintenance of pluripotency and in cell reprogramming. Indeed, it instructs the PSC-specific cell cycle, metabolism and epigenetic landscape, contributes to limit exit from pluripotency and modulates signaling cascades affecting the PSC identity. Moreover, MYC extends its regulation on pluripotency by controlling PSC-specific non-coding RNAs. In this report, we review the MYC-controlled networks, which support the pluripotent state and discuss how their perturbation could affect cell identity. We further discuss recent finding demonstrating a central role of MYC in triggering epigenetic memory in PSCs, which depends on the establishment of a WNT-centered self-reinforcing circuit. Finally, we comment on the therapeutic implications of the role of MYC in affecting PSCs. Indeed, PSCs are used for both disease and cancer modeling and to derive cells for regenerative medicine. For these reasons, unraveling the MYC-mediated mechanism in those cells is fundamental to exploit their full potential and to identify therapeutic targets.

## Pluripotent stem cells identity

Pluripotent stem cells (PSCs) possess two defining properties: they are able to indefinitely self-renew, thus maintaining their cell identity after cell division, and they are pluripotent, having the potential to differentiate toward all cell lineages of the organism. Those properties have made PSCs an attractive tool in many research areas such as developmental studies (Keller, 2005; Niwa, 2010), regenerative medicine (Cohen and Melton, 2011; Wu and Hochedlinger, 2011; Cherry and Daley, 2012), drugs discovery (Grskovic et al., 2011; Avior et al., 2016), disease and cancer modeling (Zhu et al., 2011; Bellin et al., 2012; Robinton and Daley, 2012; Kim and Zaret, 2015; Laplane et al., 2015; Zeltner and Studer, 2015; Avior et al., 2016).

In *vivo*, PSCs are a transient cell population emerging approximately at embryonic day 3.5 (E3.5) in mouse embryos, and are confined to the inner cell mass (ICM) of the blastocyst, which will further differentiate giving rise to all embryonic tissues (Cockburn and Rossant, 2010). Despite this transient existence during embryogenesis, many different PSCs have been derived and indefinitely maintained *in vitro*, including embryonic stem cells (ESCs) derived from the ICM (Evans and Kaufman, 1981; Martin, 1981; Thomson et al., 1998), post-implantation epiblast-derived stem cells (EpiSCs) (Brons et al., 2007; Tesar et al., 2007), and induced PSCs (iPSCs), obtained through the reprogramming of somatic cells (Takahashi and Yamanaka, 2006; Takahashi et al., 2007).

The in *vitro* derivation and maintenance of all those PSCs is strictly dependent on provided extrinsic signals, as PSCs continuously balance their self-renewal and differentiation potential in response to environmental cues, which are integrated with the epigenetic machinery and the transcriptional regulatory network (TRN), governing cell identity (Chen et al., 2008; Ying et al., 2008; Ng and Surani, 2011; Clevers et al., 2014; Fagnocchi et al., 2016b). Thus, to identify the molecular mechanisms which are responsible for pluripotency is fundamental to fully exploit the potential of PSCs. Our major understanding of the TRN governing pluripotency comes from studies on mouse ESCs (mESCs), which lead to the identification of the core transcription factors (TFs) required for their cell identity: Oct4 (also known as Pou5f1), Sox2 and Nanog (collectively known as OSN). Oct4 and Nanog were identified as core TFs of pluripotency due to their specific expression during early development and in ESCs, and were demonstrated to affect both the establishment and the maintenance of a stable pluripotent state both *in vivo* and *in vitro* (Nichols et al., 1998; Avilion et al., 2003; Chambers et al., 2003; Mitsui et al., 2003; Loh et al., 2006). Even if ESCs can be propagated in absence of Nanog and it is expressed at low levels in mouse EpiSCs, it is required for the formation of the ICM *in vivo* and widely co-localize with Oct4 and Sox2 in ESCs (Chambers et al., 2007; Marson et al., 2008; Silva et al., 2009). Oct4 functions as a heterodimer with Sox2 and they act sinergically, activating distal regulatory elements which control multiple pluripotency factors (Avilion et al., 2003; Masui et al., 2007). Importantly, mapped OSN targets show extensive overlap between mESCs and human ESCs (hESCs), pointing toward the existence of a conserved core TRN (Boyer et al., 2005; Loh et al., 2006). The OSN core positively regulates their own promoters, generating an interconnected auto-regulatory loop and exerts its role by concomitantly sustaining pluripotency and self-renewal factors, while restricting differentiation by repressing lineage-specificing TFs. When OSN are expressed at optimal levels, ESCs are stably maintained, while their perturbation leads to exit pluripotency and cell differentiation (Chambers et al., 2007; Toyooka et al., 2008; Karwacki-Neisius et al., 2013). Of note, an extended TRN have been elucidated in mESCs, comprising multiple TFs and downstream effectors of signaling pathways, which influence the ability of OSN to sustain PSCs identity (e.g.,: Klf4, Klf2, Dax1, Nac1, Zfp281, Essrb, Sall4, Tbx3, Prdm14, Stat3, Smad1, and Tcf3) (Niwa et al., 1998; Chen et al., 2008; Cole et al., 2008; Kim et al., 2008; Ng and Surani, 2011; Fagnocchi et al., 2016b).

Among the TFs which have been shown to play a crucial role for PSCs identity, MYC family members MYC and MYCN modulate both the establishment and the maintenance of PSCs (Chappell and Dalton, 2013). Indeed, co-deletion of both *myc* and *mycn* disrupts the maintenance of ESCs and iPSCs, while favoring their differentiation (Cartwright et al., 2005; Smith et al., 2010; Varlakhanova et al., 2010; Fagnocchi et al., 2016a). In addition MYC is essential to efficiently generate fully reprogrammed mouse and human iPSC, by enhancing OSN activity in the early steps of reprogramming (Takahashi and Yamanaka, 2006; Takahashi et al., 2007; Soufi et al., 2012). In this review, we will provide a brief overview on MYC transcription factors and then focus on the multiple mechanisms through which they can favor the pluripotent state, by integrating their transcriptional regulation activity with signaling pathways and epigenetic players. Finally, we will discuss the potential therapeutic implications of the described MYC-dependent regulatory networks.

## MYC transcription factors

MYC (also called c-MYC) was first identified more than 30 years ago as a cellular homolog of the *v-myc* oncogene of the avian myelocytomatosis retrovirus (Hayward et al., 1981; Vennstrom et al., 1982). It belongs to a basic helix–loop–helix leucine-zipper (bHLH-LZ) family of TFs comprising also MYCN and MYCL, which are evolutionarily conserved and share significant protein sequence similarities. MYC proteins comprise an N-terminal transactivation domain (TAD), several conserved motifs named MYC boxes (MBI, II, III, and IV), which have been described to be important for MYC multiple activities, a nuclear localization sequence (NLS) and a C-terminal region with the bHLH and the LZ domains, fundamental in mediating DNA binding and also protein-protein interactions. (Cole and Cowling, 2008). Generally MYC requires to heterodimerize with the bHLH-LZ protein MAX in order to bind the canonical E-box elements (CACGTG) or other non-canonical variants (CANNTG) (Blackwell et al., 1990, 1993; Blackwood and Eisenman, 1991; Solomon et al., 1993; Lin et al., 2012). MYC binding sites frequently correlate with active promoters, which are enriched for DNase I hypersensitive sites, active chromatin marks and CpG islands (Fernandez et al., 2003; Guccione et al., 2006; Zeller et al., 2006; Kim et al., 2008). This chromatin patter is well in line with the finding that MYC cannot act as a pioneer TF but rather requires an open chromatin context to access DNA, as found in iPSC (Soufi et al., 2012). More recently, ChIP-seq data demonstrated that MYC also localizes at distal regulatory elements, in a cell type and concentration-dependent manner, binding enhancer regions when highly expressed (Lin et al., 2012; Nie et al., 2012; Sabo et al., 2014; Walz et al., 2014).

At the functional level, MYC is a relatively weak transcriptional modulator, leading to small changes of its target genes. It exerts its functions by interplaying with a large set of other TFs, co-activators or by recruiting chromatin modifiers (Tu et al., 2015). MYC binding has been predominantly associated with activation of its target genes, accomplished through the recruitment of chromatin modifying factors (such as histone acetyltransferases) or by directly interacting with transcriptional co-activators (such as Mediator and P-TEFb complex), finally leading to release of stalled RNA polymerase II complex and to transcriptional elongation (Cole and Nikiforov, 2006; Zippo et al., 2007, 2009). Nonetheless, different mechanisms through which MYC mediates transcriptional repression have also been described, among which the best documented is the interaction with the transcription factor MIZ-1 (Seoane et al., 2001; Staller et al., 2001; Herkert and Eilers, 2010; Walz et al., 2014). Recently, the selective activity of MYC to transcriptionally modulate its targets has been challenged by the finding that it can eventually invade all active elements in the genome, mediating or potentiating their transcription, thus acting as a general “amplifier” (Lin et al., 2012; Nie et al., 2012). However, this phenomenon strictly depends on both the cell type and the level of expression of MYC, it does not account for genes that are repressed by MYC and it has been proposed as a secondary effect of MYC-mediated modulation of specific targets (Kress et al., 2015). In spite of the large amount of both RNA and chromatin profiling available, a unified model explaining the mechanism of MYC in modulating transcription is, indeed, still not available.

Under physiological conditions, MYC proteins have well-established pivotal roles in influencing basic cellular processes such as cell-cycle progression, cell proliferation and growth, cell size, energy metabolism, DNA replication, RNA production, differentiation and apoptosis (Eilers and Eisenman, 2008; Van Riggelen et al., 2010; Dang, 2013). For this reason, during adult tissues homeostasis, MYC expression is finely regulated by transcriptional, post-transcriptional and post-translational regulatory mechanisms (Levens, 2010; Farrell and Sears, 2014) and typically maintained at low levels or restricted to regenerating and proliferating cells (such as in epidermis and gut). On the other hand, being such a critic hub for cell regulatory networks, its over-expression is a hallmark associated to up to the 70% of all human cancers (Dang, 2012; Ciriello et al., 2013; Gabay et al., 2014). Both MYC and MYCN have a well-documented transforming capacity of cells *in vitro*, while MYCL is deficient in oncogenic potential (Land et al., 1983; Barrett et al., 1992). During tumorigenesis, over-expression of MYC is achieved either directly through gene amplification or translocation, or indirectly through mis-regulation of the many signaling pathways which are themselves targets of oncogenic mutations and regulate its expression under physiological conditions (e.g., Ras, Wnt, and Notch) (Dang, 2012). In addition to drive cancer initiation, MYC is also responsible for its maintenance: in several MYC-driven mouse tumor models, blocking MYC activity elicits tumor regression by promoting growth arrest and apoptosis and inducing cell differentiation, such as in sarcoma and hepatocellular cancer (Felsher and Bishop, 1999; Jain et al., 2002; Pelengaris et al., 2002; Shachaf et al., 2004). Finally, MYC seems also to play a crucial role in tumors driven by other oncogenes (e.g., RAS and SV40 T antigen), as targeting endogenous MYC leads to their regression (Soucek et al., 2008; Sodir et al., 2011). Altogether these evidences underline the potential of targeting MYC and its modulated targets for cancer therapy.

## Role of MYC in the maintenance of PSCS

**Figure 1:**
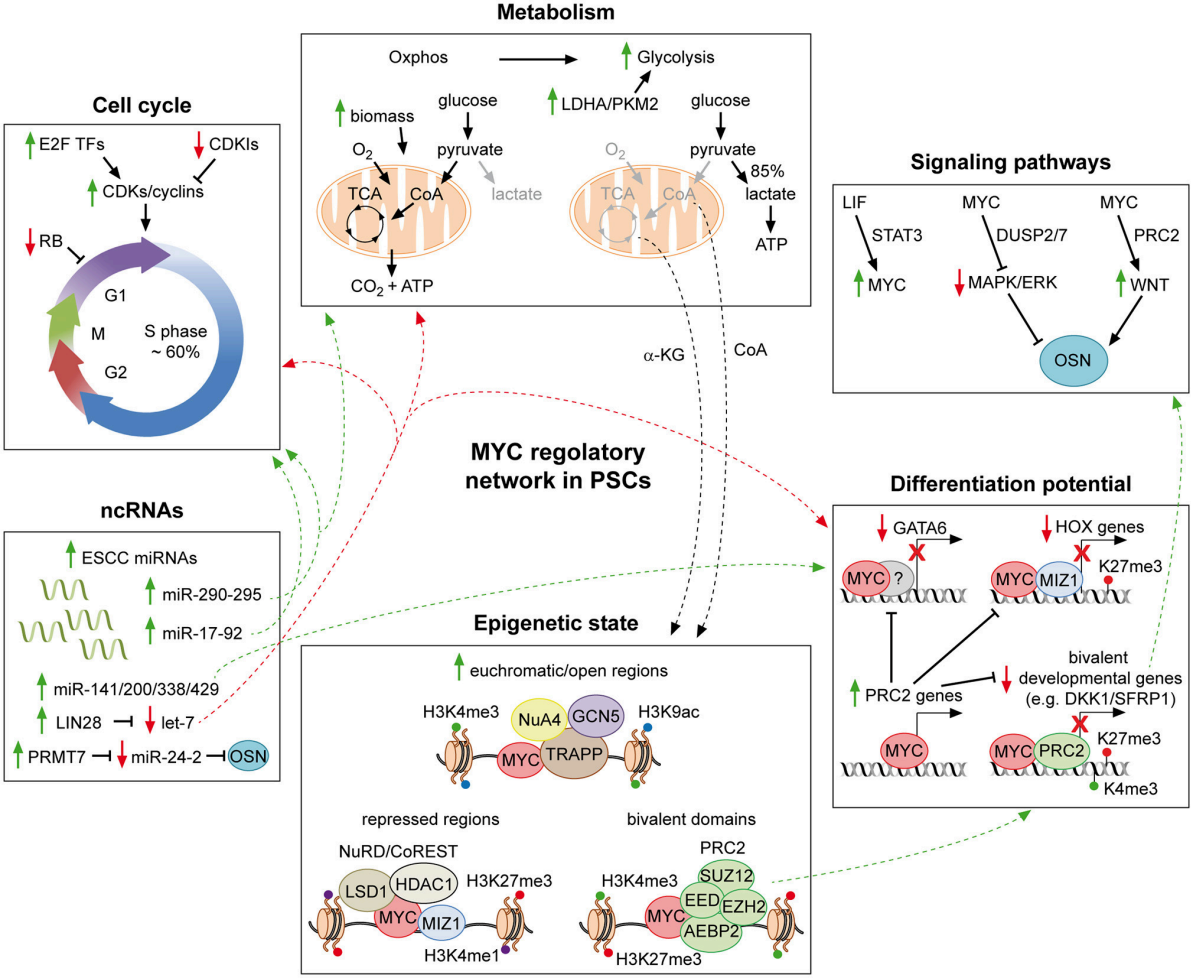
**MYC regulatory networks in pluripotent stem cells**. Schematic representation of part of the molecular mechanisms through which MYC affects the PSC identity. MYC supports the pluripotent state by favoring both the typical cell cycle structure and metabolism of PSCs. In addition, it contributes to repress developmental genes, in collaboration with MIZ-1 and PRC2. MYC further extends its regulatory action by integrating a complex network of ncRNAs, which finally affects all aspects of the PSC life. Moreover, by interacting with chromatin modifiers and remodelers, MYC controls the epigenetic state of PSCs. Finally, it is also a downstream effector of the LIF pathway and involved in modulating both the MAPK/ERK and the WNT signaling, which converge on the core of PSCs. For detailed description see the text. Solid green and red arrows indicate a MYC-mediated positive or negative regulation of nearby genes, respectively. Dotted green and red arrows are used to indicate the integration of multiple roles of MYC, which can either sustain or counteract the pluripotent state, respectively. Solid black arrows and flat lines indicate activation or repression, respectively. Red crosses indicate lack of transcription of reported genes.

MYC-mediated maintenance of PSCs relies on its central role in cellular complex regulatory networks, integrating environmental signaling pathways with transcriptional and epigenetic modulations (Figure 1). In the following sections we will discuss in details the various mechanisms through which MYC sustains PSC identity.

### MYC TFs in early embryonic development

The first evidence supporting the implication of MYC TFs in sustaining pluripotency is represented by their role during embryonic development. At the pre- and early post-implantation embryonic stages, MYC and MYCN share the same expression profiles and are functionally redundant, as MYCN can substitute MYC throughout development, leading to viable and fertile adult mice (Zimmerman et al., 1986; Malynn et al., 2000). As a consequence, single knock-out mutations on either *myc* or *mycn* do not cause developmental defects before gastrulation. In addition, pluripotent and self-renewing ESCs can be derived from ICM lacking either *myc* or *mycn*. At mid-gestation stage, instead, their expression starts to diversify, leading to abnormalities: *myc* null embryos arrest at E10.5, showing growth, cardiac, neural, hematopoietic and vascular defects, and delay or failure in turning of the embryo (Davis et al., 1993; Trumpp et al., 2001; Baudino et al., 2002; Dubois et al., 2008); *mycn* knock-out embryos die between E10.5 and E12.5, exhibiting aberrations in limb bud, visceral organs (lung, stomach, liver, and heart) and the central/peripheral nervous systems (Charron et al., 1992; Stanton et al., 1992; Sawai et al., 1993). On the contrary, MYCL is totally dispensable during the whole embryogenesis and *mycl*^−/−^ mice, expressing MYC or MYCN, are viable without abnormalities (Hatton et al., 1996).

In spite of the absence of defects related to *myc* or *mycn* single null mutants, two key works demonstrated that the expression of at least one of the two genes is mandatory during murine early embryonic development (Smith et al., 2010; Varlakhanova et al., 2010). Concomitant disruption of *myc* and *mycn* triggers spontaneous differentiation of ESCs and iPSCs toward primitive endoderm and mesoderm, and embryos injected with ESCs lacking both MYC TFs predominantly failed to generate chimeras or survived only during the early development until mid-gestation, with severe defects. Importantly, re-expression of either *myc* or *mycn* was sufficient to restore PSCs identity, while MYCL failed to complement the double knock-out defects. Likewise, single loss of either *myc* or *mycn* is inconsequential in hematopoietic stem cells, but their concurrent deletion is lethal (Laurenti et al., 2008). These evidences support the idea that MYC and MYCN are redundant but essential to determine the PSCs identity during pre-implantation stages of embryogenesis, when the ESCs of the ICM are established, whereas MYCL, although expressed in multiple organs during embryo development, is dispensable.

### The MYC module and the regulatory networks of PSCs

In the last decade, the circuitry regulating PSC identity has been deeply investigated by means of next generation sequencing, in order to identify the genomic localization of functionally relevant TFs and the transcriptional outcome of their binding (Boyer et al., 2005; Loh et al., 2006; Chen et al., 2008; Cole et al., 2008; Kim et al., 2008, 2010). These analyses were mainly performed in murine ESCs and led to the possibility to simplify their complex regulatory networks by breaking them up into distinct units, also called “modules” (Kim et al., 2010). Three units were defined in mESCs, according to the co-binding of selected factors: The core, the polycomb and the MYC modules. As stated above, the TFs belonging to the core module comprises not only the proto-typical OSN, but also other TFs and terminal components of signaling pathways (Ng and Surani, 2011; Fagnocchi et al., 2016b). The concomitant binding of multiple core TFs on both promoters and enhancers leads to the transcriptional activation of hundreds of target genes, whose expression is required for ESCs maintenance but then get turned off upon cell lineage commitment (Chen et al., 2008; Kim et al., 2008). On the contrary, the polycomb module comprises targets of the polycomb repressive complex 1 (PRC1) and PRC2, which include differentiation- and development-related genes (Bernstein et al., 2006; Boyer et al., 2006; Lee et al., 2006). Importantly, there is almost no overlap between the core and the polycomb modules, as they are involved in different functions.

The MYC module was first defined in ESCs as the common targets of MYC, MYCN, and other factors (such as MAX, DMAP1, ZFX, E2F1, E2F4, TRIM28, and CNOT) and comprised over 500 genes, which were not shared with neither the core nor the polycomb modules (Chen et al., 2008; Hu et al., 2009; Kim et al., 2010). Subsequent analysis on multiple genome wide binding studies showed that MYC binds as much as 8000 targets in ESCs, which are, indeed, clearly distinct from those regulated by either core TFs or PRC1/2 (Smith et al., 2011). This suggests that the global function of MYC in supporting PSCs identity is not solely relying on its direct binding and modulation of well-established pluripotency or lineage specification genes, but rather on its central role as integrator of multiple gene networks, which finally converge onto and influence the core module. In fact, this “enlarged MYC module” comprises genes related to functions distinctly important for the maintenance of self-renewal and pluripotency, such as cell-cycle progression, cell growth and metabolic regulation (Kim et al., 2010; Smith et al., 2011). Importantly, functionally relevant exceptions to this general activity of MYC have been described: for example MYC binds and regulates the genes coding for both SOX2 and the endoderm master regulator GATA6, thus directly affecting the core TRN and lineage differentiation, respectively (Lin et al., 2009b; Smith et al., 2010). Furthermore, MYC affects pluripotency by transcriptionally regulating different clusters of micro RNAs (miRNAs) and inhibits cell differentiation by directly and indirectly modulating developmental related genes and PRC1/2 components (Lin et al., 2009b; Smith and Dalton, 2010; Neri et al., 2012; Fagnocchi et al., 2016a). Finally, the role of MYC as a central integrator of multiple circuits in PSCs is underlined by its connection with epigenetic players and signaling pathway components, both influencing the pluripotent core module (Chen et al., 2008; Smith and Dalton, 2010; Chappell and Dalton, 2013; Fagnocchi et al., 2016a). Interestingly, the MYC module was also found to be highly expressed in cancer cells and to be predictive of cancer outcomes (Kim et al., 2010), emphasizing the potential therapeutic implications of unraveling the MYC-mediated regulatory circuitry in PSCs.

### MYC and cell cycle control

PSCs posses peculiar cell cycle duration time and structure, which differ to the ones of committed somatic cells (Figure 1, upper left panel). During embryogenesis, PSCs of the pre- and early post-implantation stages divide at unusually rapid rates (with an estimated generation time as brief as 4.4 h), to support the expansion in cell number of the developing embryo. Those rapid cell cycles comprise truncated G1/G2 gap phases and 50–60% of their time consists in DNA replication (S phase), which is alternated with rounds of chromosome segregation (M phase). On the contrary, after the first cell fate commitment during gastrulation, cell cycle is restructured and dilated to 16 h or more. At this time of development, most cells spend the majority of their time in extended G1/G2 phases (Snow, 1977; Mac Auley et al., 1993; Power and Tam, 1993; Stead et al., 2002; Faast et al., 2004). Importantly, the peculiar cell cycle pattern occurring within the ICM is recapitulated by *in vitro* cultured PSCs and is conserved among different species. (Stead et al., 2002; White et al., 2005; Fluckiger et al., 2006; Tesar et al., 2007).

The molecular mechanisms underpinning these unusual cell cycles have been investigated in mESCs. These studies showed that cyclin-dependent kinases (CDKs) are constitutively active and expressed to significantly elevated levels in mESCs with respect to committed cells, where their expression become cell cycle regulated and oscillates in a strictly periodic manner (Stead et al., 2002; Faast et al., 2004). As a consequence, the retinoblastoma tumor suppressor (RB) is constantly inactiveted due to its CDKs-mediated hyper-phosphorylation, thereby the major G1 checkpoint (the R-point) is lost and cell division is mitogen-independent (Savatier et al., 1994). In addition, CDK inhibitory molecules (CDKIs) are also not expressed in mESCs, supporting constitutive activity of CDK and rapid cell divisions (Faast et al., 2004; White et al., 2005). Despite sharing a similar cell cycle pattern with mESCs, hESCs possess slower timing of division and display cycle-dependent expression of CDKs and regulation of RB, and increased level of CDKIs, similarly to differentiated somatic cells. Thus, alternative molecular regulation of cell cycle must exist in hESCs, which have been shown to require the activity of CDK1/2 (Neganova et al., 2009, 2011, 2014).

The central role of MYC in affecting the timing and pattern of cell cycle in PSCs is underlined during early embryogenesis. Upon the first commitment of PSCs, MYC levels collapse and cell cycle structure acquires the typical somatic cell characteristics. Accordingly, genome wide binding analysis demonstrates that cell cycle regulators, such as cyclins, CDKs and E2F TFs are widely enriched among MYC targets in PSCs (Kidder et al., 2008; Kim et al., 2008; Smith et al., 2011; Nie et al., 2012; Bretones et al., 2015). Indeed, elevated MYC activity accelerates the progression toward G1 and increases the cell division rate and cell proliferation. On the contrary, concomitant *myc* and *mycn* null ESC mutants possess shortened S and lengthened G1 phases (Smith et al., 2010). Mechanistically, MYC can either directly activate transcription of several cyclins and CDKs (such as cyclin A, B1, D1/2, and E2, and CDK1, 4, 6, and 7 in PSCs) or indirectly modulate their protein stability by regulating CDK activating kinases (CAK) and CDC25 family phosphatases (Smith and Dalton, 2010; Bretones et al., 2015). In addition, MYC suppresses CDKIs, such as p21^Cip1^, p27^Kip1^ and INK4 family members, by either antagonizing MIZ-1, recruiting chromatin modifiers or transcriptionally modulating other regulators (Brenner et al., 2005; Bretones et al., 2015). Moreover, both MYC and MYCN have been demonstrated to physically interact with RB and favor the loss of the R-point cell cycle arrest (Rustgi et al., 1991; Goodrich and Lee, 1992). Finally, MYC extends its control on cell cycle regulators by modulating the expression of miRNAs clusters, as discussed below in details. This role of MYC in sustaining PSCs specific cell cycle structure is relevant to facilitate their proliferation and maintain their long term identity. Indeed, cells preferentially initiate differentiation from G1 phase, suggesting that short G1 would limit the differentiation potential of PSCs, by impeding the complex chromatin remodeling required to exit pluripotency (Filipczyk et al., 2007; Singh and Dalton, 2009; Sela et al., 2012; Pauklin and Vallier, 2013; Dalton, 2015). Importantly, PSC cell cycle peculiar traits are reminiscent of tumor cells, in which cell cycle is deregulated independently from extrinsic mitogenes.

### MYC and metabolic regulation

As it happens for cell cycle, the metabolism of PSCs also differs considerably from that of committed cells, due to different availability of oxygen and substrates which those cells experience. Somatic cells normally generate energy through carbon sources oxidation and production of reducing agents which triggers the electron transport chain (ETC) and leads to oxidative phosphorylation (Oxphos) (Figure 1, upper central panel). On the contrary, PSCs possess less mitochondrial content than differentiated counterparts and shift their metabolism to aerobic glycolysis, reminding the Warburg effect typical of highly proliferating tumor cells (Warburg, 1956; Van Blerkom, 2009; Moussaieff et al., 2015a). Despite the fact that PSCs mainly rely on glycolysis to produce energy (Zhang et al., 2012), different state of pluripotency are characterized by the use of different metabolic pathways. PSCs from the ICM at pre-implantation stage produce energy by using mainly Oxphos rather than glycolysis, and are recapitulated *in vitro* by mESCs. On the contrary, immediately after implantation, the embryo is inefficiently vascularized and oxygen availability is limited, therefore energy production relies mainly on glycolysis, similarly to the metabolic state of murine EpiSCs and hESCs (Leese, 2012; Ryall et al., 2015). Finally, recently derived naïve hESCs, resembling human ICM cells, exhibit an even higher glycolytic flux accompanied by Oxphos (Carbognin et al., 2016; Gu et al., 2016). The reason why different PSCs utilize diverse metabolic pathways is still debated. Importantly, recent findings point toward the idea that cellular metabolism is not only a mere consequence of the cellular environment but rather is an active mechanism affecting PSCs identity in multiple ways. In addition to generating energy, metabolism supports PSCs identity by providing both the molecular intermediates required to sustain rapid cellular divisions and growth (Vander Heiden et al., 2009; Lunt and Vander Heiden, 2011) and metabolites influencing their epigenome (Shyh-Chang et al., 2013; Shiraki et al., 2014; Carey et al., 2015; Moussaieff et al., 2015a,b).

In this sense, the well-documented role of MYC in modulating metabolic related enzymes, energy production and biomass accumulation assume particular relevance for its ability to support PSCs identity. MYC regulates the glucose metabolism, by stimulating virtually all genes involved in both glycolysis and glutaminolysis, it favors the synthesis of nucleotides, proteins and lipids, thus supporting cell growth and proliferation and, finally, it is involved in mitochondrial and ribosomal biogenesis (Dang, 2013; Stine et al., 2015). Recent evidences underline the importance of MYC in regulating the glycolitic metabolism of both mouse and human ESCs (Cao et al., 2015; Gu et al., 2016). Cao et al. (2015) demonstrated that, when MBD2-mediated repression of MYC is alleviated by the miR-290 cluster of miRNAs, it directly binds to the promoter of two key relevant glycolytic enzymes, namely PKM2 and LDHA, activating their expression and enhancing the glycolytic metabolic switch of mESCs. Accordingly *myc*^−/−^ mESCs show diminished glycolityc signature. The same molecular mechanism seems to be conserved in humans and favors the reprogramming of human fibroblasts. Gu et al. (2016), instead, showed that an increased glycolytic flux, functionally relevant to support the PSC state, occurs during the transition from naïve to primed hESCs and this correlates with augmented MYC transcriptional activity and elevated levels of nuclear MYCN. In particular, MYC-driven glycolysis depends on MCT1, a monocarboxylates membrane transporter. In accordance with this, chemical inhibition of either MYCN or MCT1 results in decreased proliferation of naïve hESCs. Furthermore, in addition to directly modulating the metabolism, MYC is also hard-wired into miRNAs networks affecting metabolic pathways, as discussed below. Finally, several recent works demonstrate how metabolic intermediates, such as α-ketoglutarate (α-KG), S-adenosylmethionine (SAM), acetyl-coenzyme A (CoA), nicotinamide adenine dinucleotide (NAD), and flavin adenine dinucleotide (FAD) influence histone acetylation and DNA/histone methylation in PSCs, thus regulating their maintenance or differentiation (Shyh-Chang et al., 2013; Shiraki et al., 2014; Carey et al., 2015; Ryall et al., 2015; Moussaieff et al., 2015a,b). MYC-mediated modulation of processes such as glycolysis and glutaminolysis, which alters the availability of α-KG and CoA, suggests that it may also affect the epigenome of PSCs as a consequence, even though this hypothesis has to be formally proven yet.

### MYC regulation of the differentiation potential of PSCs

PSCs identity depends on the continuous balance between their self-renewal and differentiation potentials. While the core TFs reinforce the expression of pluripotency genes, they also contribute to repress lineage-specific regulators, thus preventing the exit from the pluripotent state. OSN bind to and repress developmental-related genes in combination with repressive chromatin modifiers, such as SETDB1 and polycomb group (PcG) proteins, in both mouse and human ESCs (Boyer et al., 2006; Bracken et al., 2006; Lee et al., 2006; Young, 2011). Consequently, the loss of OSN-mediated repression rapidly leads to induction of developmental genes and to lineage commitment (Bernstein et al., 2006; Mikkelsen et al., 2007; Pan et al., 2007; Zhao et al., 2007).

Similarly, MYC TFs do not maintain PSCs identity only by regulating cell cycle, metabolic genes and cell growth/proliferation, as discussed above, but also by inhibiting PSCs differentiation, as demonstrated by several lines of evidence (Figure 1, lower right panel). In 2010, Smith and colleagues demonstrated that the simultaneous double knock-out of both *myc* and *mycn* (dKO) in either mESCs or iPSCs results in their differentiation toward primitive endoderm (Smith et al., 2010). This was attributed to the MYC-mediated repression of GATA6, a master regulator of primitive endoderm lineage commitment (Fujikura et al., 2002). Importantly, re-expression of either *myc* or *mycn* in the dKO background was sufficient to restore pluripotency, suggesting that the two TFs act redundantly in repressing GATA6 in PSCs. In addition to GATA6, MYC was also found to bind HES1 and transcriptionally repress FOXA2 and SOX17, other important primitive endoderm markers, while, in another report, it was demonstrated to limit hematopoietic differentiation, by repressing lineage-specific transcriptional activators and differentiation markers (Smith et al., 2010; Varlakhanova et al., 2010). These evidences suggest a more broad activity of MYC in regulating the differentiation potential of PSCs. Accordingly, MYC acts coordinately with MIZ-1 in suppressing differentiation of hESCs, by targeting and repressing a broad range of developmental related genes, including HOX clusters. MYC-mediated repression is less characterized compared to its positive regulatory activity, nonetheless, it is known that MYC is able to bind to MIZ-1 targets and inhibit its transcriptional activation. In addition, the MYC/MIZ-1 complex acts as a repressor by also recruiting histone deacetylases (HDACs) and DNA methyltransferases (DNMTs), which promote gene silencing (Herkert and Eilers, 2010). In hESCs, ~200 genes are co-bound by MYC/MIZ-1 comprising HOX and genes involved in neural and muscle differentiation, and at least 36 HOX genes were proven to be transcriptionally down-regulated upon MYC disruption (Varlakhanova et al., 2011). Importantly, the MYC/MIZ-1 co-bound developmental genes are enriched in H3K27me3 histone mark, suggesting a potential link between MYC and PcG in PSCs. In line with this hypothesis, genome wide analysis reveals that ~10% of MYC targets in mESCs are shared with the PRC2 protein SUZ12, leading to modulation of HOX, ectoderm and neural development-related genes (Lin et al., 2009b). More recently, we aimed to discern the functional roles of MYC in sustaining PSCs identity, by comparing mESCs grown in their standard culture medium, containing leukemia inhibitory factor (LIF) and serum, with cells grown solely in MYC-dependency (Fagnocchi et al., 2016a). To this end, we used mESCs expressing a MYC^T58A^-estrogen receptor (MYC-ER) fusion protein, activated by 4-hydroxytamoxifenm (OHT) (Cartwright et al., 2005; Fagnocchi et al., 2016a). Importantly, we found that MYC down-regulates developmental-related genes involved in multi-lineage commitment which are targeted by PRC2 and/or PRC1. Almost half of total genes down-regulated by MYC in mESCs correlate with genes repressed by PRC2, and, for a subset of those genes (such as Dkk1, Sfrp1, Sfrp5, and Apcdd1) we demonstrated the direct role of MYC in recruiting the PRC2 complex on their promoters to mediate their transcriptional repression. Both MYC and MYCN physically interacts with PRC2 in mESCs. Indeed, thanks to biochemical approaches, we demonstrated that MYC, together with MAX, interacts with the three core component of PRC2 (Eed, Ezh2, and Suz12) and the Aebp2 associated protein, through its conserved MBII motif located in N-terminal TAD. Functional analysis revealed that the MYC/PRC2-induced inhibition of developmental pathways is required to maintain mESCs identity, as the knocking-down of either Eed or Ezh2, leads to spontaneous differentiation of MYC-dependent cells (Fagnocchi et al., 2016a). Altogether, these findings demonstrate the global role of MYC in modulating the differentiation potential of PSCs, in association with other TFs and chromatin players.

In addition to MYC-mediated direct modulation, it indirectly limits multi-lineage development of mESCs by transcriptionally up-regulating all genes coding for the PRC2 and associated factors (Neri et al., 2012). Furthermore, MYC is integrated in a regulatory circuitry including multiple miRNAs, which regulates expression of developmental-related genes in PSCs, as commented in the next section.

### MYC integration with non-coding RNAs regulatory circuits

Non-coding RNAs (ncRNAs) are primary players in defining PSCs identity (Greve et al., 2013; Rosa and Brivanlou, 2013; Rosa and Ballarino, 2016). Among them, miRNAs are ~20-base long, processed and single stranded transcripts, which recognize mRNAs by base pairing of a short “seed” sequence at their 5′end, thus leading to translational inhibition or degradation of their targets (Fabian and Sonenberg, 2012). Evidence for their role in pluripotency was first derived from mESCs lacking either Dicer or Dgcr8, two key factors in miRNAs maturation. Dicer- or Dgcr8-deficient mESCs are viable, but greatly impaired in cell cycle, proliferation and unable to properly differentiate (Kanellopoulou et al., 2005; Murchison et al., 2005; Wang et al., 2007). A specific family of miRNAs, sharing the same AAGUGC seed sequence, are conserved and majorly expressed among PSCs, the so called ESC-specific cell-cycle regulating (ESCC) miRNAs, which, together with ESCC-like miRNAs (shifted by one base), comprise up to the 60% of all the miRNA population in PSCs. This family includes the clusters miR-290–295 and miR-17–92b in mESCs and miR-302–367 in mEpiSCs and hESCs, which, with exception of miR-17–92b, are not expressed in somatic cells. Conversely, the let-7 family of miRNAs is post-transciptionally repressed by the RNA binding protein LIN28, which is specifically expressed in PSC and down-regulated upon differentiation. (Greve et al., 2013; Rosa and Brivanlou, 2013). Both families of miRNAs play a central function in regulation of PSCs identity, underlined by the fact that their specific expression is controlled by the core TRN and they integrate with the OSN regulatory circuitry (Barroso-Deljesus et al., 2008; Card et al., 2008; Marson et al., 2008). In addition, ESCC influence PSCs by regulating their cell cycle, proliferation and differentiation potential.

Long (> 200 nucleotides) non-coding RNAs (lncRNAs) resemble protein-coding mRNAs and are usually spliced, polyadenylated and possess a 5′cap. They can either act as antisense transcripts, modulate the activity of chromatin modifiers or serve as “sponges” for miRNAs, thus affecting their activity (Guttman and Rinn, 2012). The signature of both mouse and human ESCs have been deeply investigated, identifying ESC-specific lncRNAs, which are partly targets of the core TFs and whose levels decrease upon differentiation. Accordingly, their down-regulation results in reduction of proliferation and pluripotency markers expression, in parallel with induction of lineage specific genes and differentiation (Dinger et al., 2008; Guttman et al., 2009, 2010, 2011; Loewer et al., 2010; Sheik Mohamed et al., 2010; Ng et al., 2012).

As mentioned in the previous sections, the role of MYC in maintaining the PSC state is greatly interconnected with regulation of ncRNAs (Figure 1, lower left panel). MYC modulates both the counteracting ESCC and let-7 families of miRNAs in PSCs. The ESCC miRNAs were defined with the discovery that the miR-290–295 cluster was able to rescue the Dgcr8^−/−^-induced defects on cell cycle and proliferation in mESCs. The miR-290–295 favors the rapid transition from G1 to S phases and the peculiar cell cycle structure of PSCs, by regulating the Cdk2-cyclin E complex. Importantly, miR-290–295 acts downstream of MYC, which binds their promoters, and is also involved in establishment of iPSCs, being able to replace MYC in the reprogramming of mouse fibroblasts (Wang et al., 2008; Judson et al., 2009). On the other hand, the let-7 family promotes differentiation and MYC, by transcriptionally inducing the let-7 repressor LIN28, together with OSN, limits its expression, thus supporting the PSCs state. Additionally, in lymphoma cells, MYC directly binds let-7 and represses it, while, in turn, mature let-7 inhibits MYC expression, suggesting that these two factors are involved in a double-negative feedback regulatory loop (Chang et al., 2008, 2009; Marson et al., 2008; Melton et al., 2010; Zhong et al., 2010). Importantly, the LIN28/let-7 axis has been also demonstrated to be a central regulator of PSCs metabolism, in particular as far as mitochondrial respiration is concerned (Shyh-Chang and Daley, 2013; Psathas and Thomas-Tikhonenko, 2014), suggesting MYC implication in the regulation of the Oxphos metabolism critical to ESCs (Wang et al., 2009a; Zhang et al., 2011; Carbognin et al., 2016). Furthermore, MYC induces the miR-17–92 cluster in mESCs, which supports cell cycle progression by the inhibition of controllers such as p21, cyclin D1 and RB2. Accordingly, in MYC/MYCN dKO mESCs, the miR-17–92 cluster is down-regulated and cells differentiate and re-structure their cell cycle (Smith and Dalton, 2010; Smith et al., 2010). MYC also directly activates miR-141, miR-200, miR-338, and miR429, which repress differentiation pathways (Lin et al., 2009a; Psathas and Thomas-Tikhonenko, 2014). Importantly, up-regulation or inhibition of those classes of miRNAs result in reduction or enhancement of differentiation of mESCs, respectively, thus showing the functional importance of this MYC/miRNAs integration in regulating the differentiation potential of PSCs (Lin et al., 2009a). More recently, instead, MYC has been demonstrated to indirectly limit the expression of miR-24-3p/miR-24-2-5p, which inhibit OSN and MYC itself, acting as anti-pluripotent miRNAs in mESCs. MYC and OSN activate the protein arginine methyltransferase 7 (PRMT7), which, in turn limits miR-24-3p/miR-24-2-5p expression (Lee et al., 2016). Interestingly, MYC induces a stem-cell like miRNA signature in aggressive hepatoblastomas, characterized by repression of miR-100/let-7a-2/miR-125b-1 and activation of miR-371 (part of the human homolog of murine miR-290-295), suggesting that MYC-mediated regulation of miRNAs in PSCs is partly shared with cancer cells and functionally relevant for tumorigenesis. In support of this hypothesis, reversal of the MYC-driven miRNA signature results into smaller tumor formation in mice (Chang et al., 2008; Cairo et al., 2010). Finally, MYC has also been implicated in the regulation of lncRNAs controlling pluripotency and differentiation, either by directly binding their promoters or by modulating their expression in collaboration with Dicer and miRNAs, suggesting an even broader role of MYC in controlling ncRNA in PSCs (Guttman et al., 2011; Zheng et al., 2014).

### MYC and the epigenetic state of PSCs

PSCs are endowed with unique chromatin and epigenetic organization which allow their genome to respond to two opposing needs: on one hand, it must be plastic to be ready to enter developmental pathways; on the other side, it must retain a cellular memory that specifies the PSCs state. To this end, the PSC chromatin is highly dynamic and accessible, when compared to committed cells, with large regions of transcribed euchromatin and restricted PcG-repressed and heterochromatic domains, marked with H3K27me3 and/or H3K9me3 (Brenner et al., 2005; Meshorer and Misteli, 2006; Efroni et al., 2008; Gaspar-Maia et al., 2011; Zhu et al., 2013; Tee and Reinberg, 2014). In addition, the most distinctive epigenetic feature of PSCs is represented by the presence of bivalent domains, carrying both activating H3K4me3 and repressing H3K27me3 histone modifications and marking poised developmental-related genes, ready to be activated in response to pro-differentiative stimuli (Bernstein et al., 2006; Boyer et al., 2006; Lee et al., 2006; Mikkelsen et al., 2007; Pan et al., 2007; Zhao et al., 2007).

MYC has been described to influence the global cellular chromatin structure and to favor widespread active euchromatin, by promoting hyperacetylation and modulating both H3K4me3 and H3K9ac, thus suggesting its role in defining the peculiar chromatin state of PSCs (Knoepfler et al., 2006; Frye et al., 2007; Cotterman et al., 2008) (Figure 1, lower central panel). In line with this idea, MYC null mESCs show a global epigenetic remodeling, with a decrease of ~70% of the active histone mark H3K4me3, which significantly overlap with MYC binding sites (Lin et al., 2009b). Mechanistically, MYC affects the chromatin structure and the epigenetic landscapes in multiple ways. First of all, it may transcriptionally modulate various chromatin regulators. Indeed, components of the PRC1/2, of histone acethyl- and methyltransferases complexes and of the Swi/Snf remodeling factors were identified among MYC targets in mESCs (Kidder et al., 2008; Kim et al., 2008; Neri et al., 2012; Krepelova et al., 2014). Secondly, MYC is well-documented to interact with and recruit onto the chromatin several players implicated in chromatin modifications and dynamics (Hann, 2014; Tu et al., 2015). In particular, MYC interacts with various histone acethyltransferase (HAT) complexes on their common targets, suggesting its role in modulating the global histone H3 and H4 acetylation state of PSCs (Mcmahon et al., 2000; Frank et al., 2001, 2003; Fazzio et al., 2008; Lin et al., 2009b; Kim et al., 2010). More recently, MYC has been found to co-localize on active genes with two histone demethylases (HDMs), namely Kdm4b/c, required for mESCs self-renewal, suggesting MYC implication also in the regulation of the histone methylation state of PSCs (Das et al., 2014). Furthermore, MYC may regulate transcriptional repression in PSCs by physically interacting with epigenetic regulators, such as Hdac1/3, Sin3A/B and Lsd1 (Kdm1a), which are part of chromatin repressive complexes affecting both histone acetylation and demethylation. (Liang et al., 2008; Wang et al., 2009b; Smith et al., 2011; Hu and Wade, 2012; Mcdonel et al., 2012; Amente et al., 2013; Garcia-Sanz et al., 2014). In line with these evidences, in mESCs grown in MYC-dependency, MYC interacts with PRC2 and this interaction is functionally relevant in affecting the epigenome and the identity of mESCs. Indeed, we demonstrated a global increment of both Suz12 binding and H3K27me3 deposition on promoters of bivalent genes, compared to ESCs grown in presence of LIF. In addition, MYC is able to directly recruit PRC2 on a subset of these bivalent promoters, thus augmenting H3K27me3 and repressing gene expression. Accordingly, impaired expression of either Eed or Ezh2 is not compatible with maintenance of MYC-dependent mESCs (Fagnocchi et al., 2016a).

### MYC modulation of signaling pathways

The TRN of PSCs is continuously targeted by downstream effectors of environmental signaling pathways, which, therefore, play a pivotal role in determining different pluripotent states. As a consequence, propagation of diverse PSCs strictly relies on well-defined growth conditions. Mouse ESCs were first derived on mitotically inactive embryonic fibroblasts as feeders and in presence of serum (Evans and Kaufman, 1981; Martin, 1981). Thereafter, LIF and the bone morphogenetic protein 4 (BMP4) were identified as key ingredients to propagate mESCs in feeder-and serum-free conditions, thanks to their ability to activate the Janus kinase–signal transducer and activator of transcription 3 (JAK/STAT3) pathway and repress developmental processes through the SMAD/IDs cascade, respectively (Smith et al., 1988; Williams et al., 1988; Ying et al., 2003). Nonetheless, LIF/serum medium is commonly used as standard growing condition for mESCs. Consequently, several other defined culturing media, based on the utilization of chemical inhibitors, were developed to propagate mESCs in a more “naïve” state of pluripotency (also known as “ground state”), thus being more similar to early pre-implantation PSCs of the ICM (Weinberger et al., 2016). Among them, the two inhibitors (2i)/LIF medium relies on the chemical repression of the MAPK/ERK kinases signaling, achieved with PD0325901 (PD03), and concomitant activation of the WNT pathway, through the use of CHIR99021 (CHIR), which inhibits GSK3β, thus stabilizing β-catenin, which ultimately counteracts Tcf3 pro-differentiative activity in the nucleus (Cole et al., 2008; Ying et al., 2008). Activation of the MAPK/ERK cascade drives the transition toward primed pluripotency and, therefore, both primed hESCs and murine EpiSCs are cultured in presence of basic fibroblast growth factor (FGF2), which activates MAPK/ERK, and Activin A, which reinforces the transforming growth factor-β (TGFβ) signaling (Thomson et al., 1998; Brons et al., 2007; Tesar et al., 2007). More recently, several reports identified culture conditions to propagate naïve hESCs, suggesting that this pluripotent state, similarly to its murine counterpart, relies on ablation of the MAPK/ERK pathway, activation of the BMP4, LIF, and WNT signaling cascades and low activation of Activin A/TGFβ1 (Cartwright et al., 2005; Gafni et al., 2013; Theunissen et al., 2014; Ware et al., 2014). Finally, to support the importance of LIF, BMP4, and WNT signaling pathways in determining naïve PSC identity, downstream effectors such as STAT3, SMAD1, and TCF3, respectively, are hard-wired into the core TRN (Chen et al., 2008; Cole et al., 2008; Ho et al., 2011).

**Figure 2:**
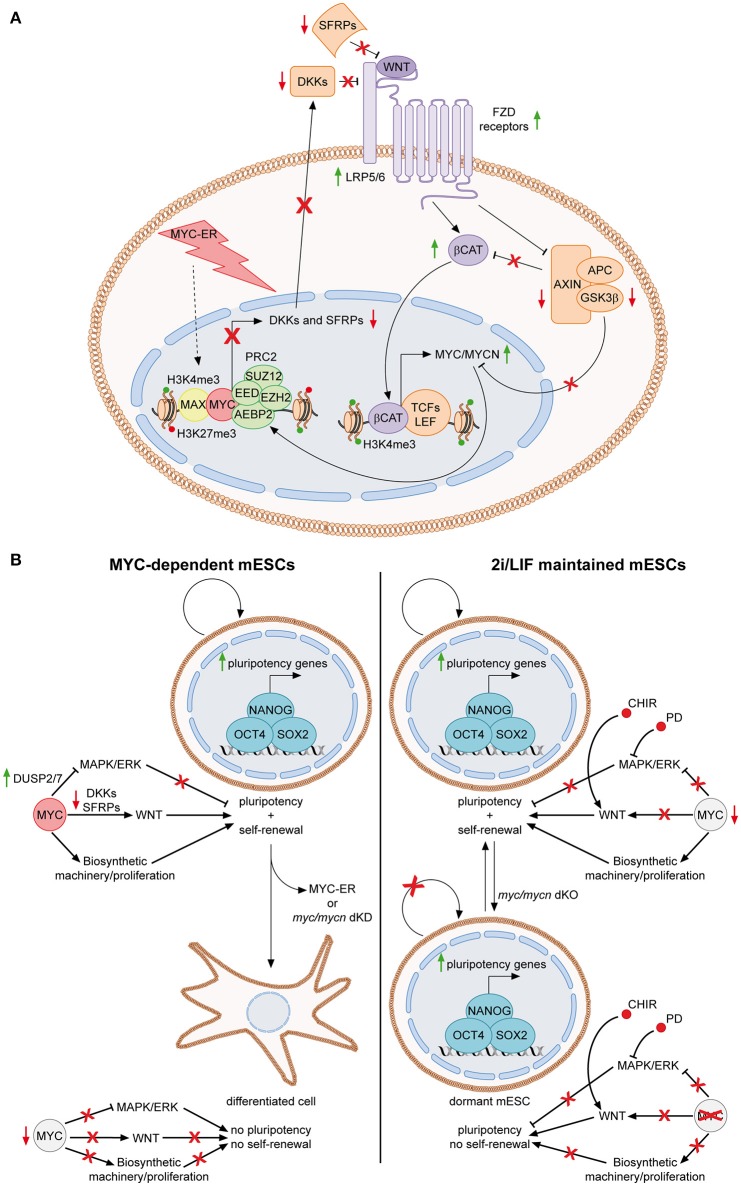
**The MYC-driven self-reinforcing loop in ESCs. (A)** MYC-dependent ESCs are maintained in presence of OHT, which activate MYC-ER, finally leading to its nuclear localization. In the nucleus, MYC-ER stimulates a self-reinforcing positive feedback loop. Together with PRC2, MYC represses antagonist of the WNT pathway, such as DKK and SFRP family members, thus resulting in activation of the WNT pathway. Consequently, active β-catenin translocates into the nucleus, where, among its targets, it transcriptionally activates both *myc* and *mycn*. Endogenous MYC and MYCN, in turn, reinforce this mechanism by contributing to the repression of WNT antagonists. The feedback positive loop is also sustained by the fact that MYC mediates the inhibition of the destruction complex (AXIN, APC, GSK3β) function, resulting in reduction of GSK3β-dependent degradation of both MYC/MYCN and β-catenin (Fagnocchi et al., 2016a). **(B)** Role of MYC in sustaining mESCs identity in MYC-dependent vs. 2i/LIF maintained cells. MYC-dependent cells rely on MYC for the concomitant down-regulation of MAPK/ERK and up-regulation of WNT pathways (Chappell et al., 2013; Fagnocchi et al., 2016a). In addition, it also sustains proliferation by inducing the biosynthetic machinery. Once MYC activity is lost, by either knocking down *myc/mycn* or by inactivating MYC-ER after short-term exposure to OHT, mESCs differentiate spontaneously as both the proliferation and the maintenance of pluripotency gene expression are affected (Cartwright et al., 2005; Fagnocchi et al., 2016a). On the contrary, in 2i/LIF cultured cells, chemical inhibitors bypass the role of MYC in modulating signaling pathways. Indeed, CHIR and PD activate and inhibit WNT and MAPK/ERK pathways, respectively (Cole et al., 2008; Ying et al., 2008). Accordingly, MYC is less expressed in 2i/LIF mESCs (Marks et al., 2012). Nonetheless, MYC still have a key function in 2i/LIF medium, as its deletion leads to impairment of the biosynthetic machinery and entrance of ESCs in a reversible dormant state (Scognamiglio et al., 2016). Concomitantly, the pluripotency is not affected as chemical inhibitors still modulate signaling to the core TRN even in the absence of MYC. Solid green and red arrows indicate high or low expression on nearby genes, respectively. Solid black arrows and flat lines indicate activation or repression, respectively. Red crosses indicate inhibition of related functions.

MYC was first connected to the signaling pathways of PSCs, when it was discovered to be a downstream target of the LIF/Stat3 cascade in mESCs (Cartwright et al., 2005) (Figure 1, upper right panel). Upon LIF withdrawal, MYC levels decrease, due to both lack of transcriptional activation and post-translational GSK3β-dependent phosphorylation of threonine 58 (T58). Importantly, sustained MYC activity, through activation of a MYC-ER fusion protein, is able to circumvent LIF-dependency of mESCs, suggesting that the LIF signaling importance for the pluripotent state is, at least partially, due to MYC functions. Accordingly, expression of a MYC dominant negative mutant leads to loss of self-renewal and differentiation (Cartwright et al., 2005). Consequently, MYC was also found to be involved in limiting the MAPK/ERK signaling, thus sustaining pluripotency of mESCs (Chappell et al., 2013). While ERK is known to stabilize MYC protein, it has been shown that MYC and MAX transcriptionally activate the dual specific phosphatases 2 and 7 (DUSP2/7), which in turn bind and inactivate ERK1/2 through its de-phosphorylation, leading to attenuation of the MAPK/ERK activity. In accordance, loss of MYC leads to down-regulation of DUSP2/7, stimulation of the MAPK/ERK pathway and differentiation of mESCs (Sears et al., 2000; Chappell et al., 2013). More recently, we demonstrated that MYC also sustains the WNT pathway in mESCs and that this is required for MYC-mediated maintenance of both self-renewal and pluripotency (Fagnocchi et al., 2016a) (Figure 2A). Indeed, MYC leads to an accumulation of nuclear β-catenin, while MYC/MYCN dKO mESCs show reduced amount of active β-catenin, respect to wild type cells. Importantly, WNT pathway activation is fundamental for MYC-mediated maintenance of mESCs, as either knocking-down β-catenin or down-modulation of WNT signaling with soluble inhibitors Dkk1 and Srfp1, result in loss of stemness and spontaneous differentiation. Mechanistically, MYC activates WNT signaling by transcriptionally activating receptors and co-receptors (such as Fzd2, 3, and 7 and Lrp5), while repressing antagonists of the pathway (such as Dkk and Sfrp inhibitors family members), by directly recruiting PRC2 on their promoters (Figure 2A). Of importance, endogenous *myc* and *mycn* genes are targets of nuclear β-catenin and MYC-mediated activation of the WNT pathway leads to their transcriptional up-regulation, in a positive self-reinforcing regulatory loop (Figure 2A) (Fagnocchi et al., 2016a).

Alltogether, these evidences collectively indicate that MYC sustains PSC identity by inducing the WNT-pathway, while limiting the MAPK/ERK signaling, resembling the activity of chemical inhibitors of the 2i/LIF culturing medium (Figure 2B). In accordance with this finding, in 2i/LIF mESCs the level of MYC expression is lower compared to LIF/serum maintained cells (Marks et al., 2012), suggesting that PD03 and CHIR bypass the requirement of MYC to modulate signaling pathways relevant for maintenance of pluripotency. While the chemicals in 2i/LIF medium may render “ground state” mESCs partly independent to MYC for their maintenance, they still require its activity for their biosynthetic homeostasis. In fact, recent findings show that pharmacological inhibition of MYC activity or MYC/MYCN concomitant deletion in mESCs cultured in 2i/LIF, results in reduced RNA processing, ribosome biogenesis, protein synthesis and, ultimately, arrest of proliferation and entering in a dormant state, remindful of diapause balstocysts (Scognamiglio et al., 2016). These conclusions suggest that MYC-mediated control of the biosynthetic machinery in 2i/LIF cultured mESCs is uncoupled from regulation of their pluripotency and rather mostly implicated in their self-renewal potential (Figure 2B).

### MYC in the reprogramming of somatic cells

Despite no data are available to prove a role for MYC in establishing PSCs *in vivo*, the major evidence underlying its function in the induction of the pluripotent state is its well-known role in reprogramming of somatic cells. MYC is one of the four transcription factors, together with OCT4, SOX2 and KLF4 (OSKM), initially identified by Shinya Yamanaka's lab as sufficient to reprogram differentiated cells to iPSCs, both in mice and humans (Takahashi and Yamanaka, 2006; Takahashi et al., 2007). Since then, many different variations from the OSKM cocktail have been used to improve the derivation of iPSCs (Buganim et al., 2013; Theunissen and Jaenisch, 2014; Takahashi and Yamanaka, 2016), nonetheless the reprogramming process in absence of MYC is generally less efficient, both in terms of the number of iPSCs and the time required to reach full reprogrammed state (Nakagawa et al., 2008; Wernig et al., 2008). Moreover, among the OSKM factors, MYC promotes the most ESC-like transcriptional signature in fibroblasts, when expressed alone (Sridharan et al., 2009).

Reprogramming is a multi-step event, throughout which MYC exerts its function since the early stages (Mikkelsen et al., 2008; Sridharan et al., 2009; Soufi et al., 2012). In fact, MYC has been proposed to mediate early global epigenetic changes, required for the formation of euchromatin. Accordingly, two HDAC inhibitors, the valproic acid (VPA) and trichostatin A (TSA), enable reprogramming of both human and murine fibroblasts in absence of MYC, indicating its role in controlling global histone acetylation during generation of iPSCs (Huangfu et al., 2008; Araki et al., 2011). These global epigenetic changes are reflected in remodeling of gene expression patterns: MYC enhances early reprogramming by repressing fibroblast specific genes more efficiently than OCT4, SOX2, and KLF4 (OSK), while inducing a PSC-like gene expression profile (Mikkelsen et al., 2008; Sridharan et al., 2009). Of importance, MYC binds to enhancers of pluripotency-related genes in early phases of de-differentiation and there it increases the OSK engagement with chromatin and its activating function. Nonetheless, it is noteworthy that MYC cannot act as a “pioneer factor” and requires previous binding of OSK to access the chromatin (Soufi et al., 2012).

Several factors can replace MYC function during reprogramming, including metabolic modulators, miRNAs and other pluripotent TFs, indicating that those mechanisms by which MYC maintains PSCs, might also be relevant for establishment of iPSCs (Yu et al., 2007; Judson et al., 2009; Choi et al., 2011; Psathas and Thomas-Tikhonenko, 2014; Cao et al., 2015; Ryall et al., 2015). In addition, the role of MYC in modulating the cell cycle might be relevant for later stages of reprogramming, as the typical cell cycle structure of PSCs is not present in partially reprogrammed cells (Singh and Dalton, 2009).

Importantly, the clinical applications of iPSCs, are partly hindered by the fact that MYC activation leads to oncogenic transformation and animals derived from iPSCs induced by OSKM frequently develop tumors (Okita et al., 2007). While poor reprogramming is achieved without MYC (Nakagawa et al., 2008; Wernig et al., 2008), the possibility to derive iPSCs with MYCL, which posses less transformation capability, and, above all, with transformation-defective MYC mutants, it is worth further investigation to obtain clinically safe reprogrammed pluripotent cells with high efficiency (Nakagawa et al., 2008, 2010).

### MYC and epigenetic memory

The epigenetic memory can be defined as “a heritable change in gene expression or behavior, that is induced by a previous developmental or environmental stimulus and cannot be explained by changes in the DNA sequence” (D'urso and Brickner, 2014). During development, cells progressively reduce their differentiation potential and acquire a committed cell identity, in response to specific stimuli, which will be then maintained long after the stimulus is passed (Ringrose and Paro, 2004; Bonasio et al., 2010). This phenotypic stability of differentiated cells relies on mechanisms which ensure the transmission of epigenetic and, consequently, transcriptional patterns. Accordingly, efficient reprogramming of committed cells toward pluripotency requires the erasure of the existing somatic epigenetic memory, which constitutes well-known barriers to formation of iPSCs (Mikkelsen et al., 2008; Pasque et al., 2012; Chen et al., 2013; Gaspar-Maia et al., 2013; Sridharan et al., 2013; Nashun et al., 2015). Mechanistically, epigenetic inheritance can be achieved by either self-propagating *trans*-acting regulatory factors, maintained by feedback loops and TF networks, or by *cis*-acting epigenetic signatures, such as DNA methylation or histone modifications, which must be maintained or re-established during and after cell division, respectively (Bonasio et al., 2010; Moazed, 2011; Wang and Higgins, 2013). Importantly, recent evidences indicate that epigenetic memory is also maintained in ESCs, even though with different mechanisms respect to somatic cells. Hence, the pluripotent state is characterized by a highly stable epigenome (Shipony et al., 2014).

**Figure 3:**
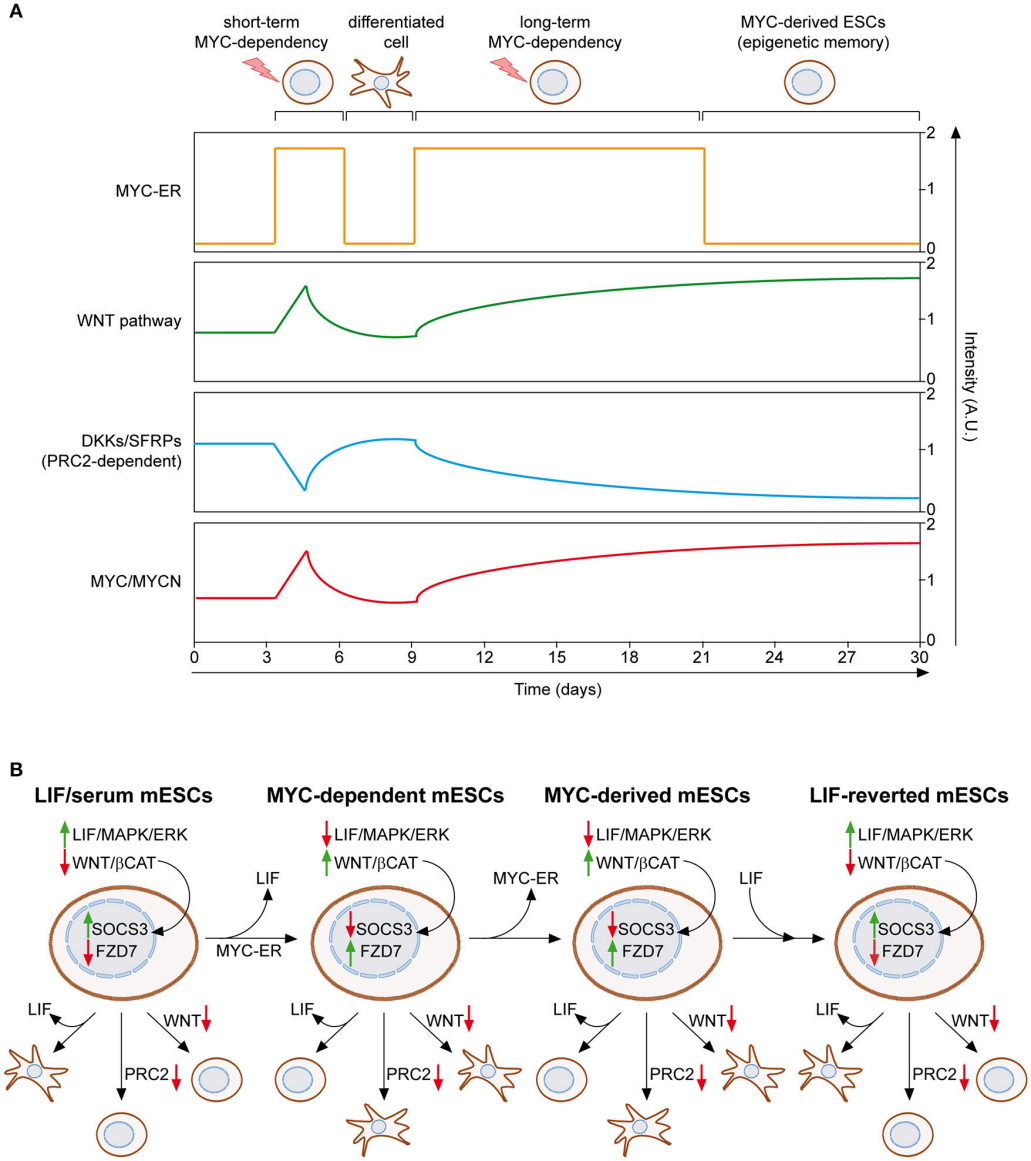
**MYC induces and epigenetic memory mechanism in ESCs. (A)** Mouse ESCs can be maintained upon LIF withdrawal, providing activation of MYC-ER, which leads to WNT pathway reinforcement, through PRC2-mediated repression of its antagonist, and induction of endogenous MYC and MYCN. If MYC-ER remains activate for short term (e.g., 3–6 days), mESCs differentiate spontaneously once it is inactivated. On the contrary, long term activation of MYC-ER (at least 12 days) leads to the stabilization of WNT reinforcement and concomitant induction of MYC/MYCN, even in the absence of the original stimulus (OHT), thanks to a self-reinforcing loop. This epigenetic memory mechanism permits the maintenance of MYC-derived mESCs in absence of both LIF and MYC-ER activation (Fagnocchi et al., 2016a). **(B)** The epigenetic memory of MYC-derived mESCs permits their reversion to a LIF-dependent state of pluripotency. LIF/serum maintained mESCs are dependent on LIF/JAK/STAT cascade; hence LIF withdrawal leads to their differentiation. On the contrary, inhibition of both PRC2 activity and WNT pathway do not affect their pluripotent state. Both MYC-dependent and MYC-derived mESCs are, instead, insensitive to LIF withdrawal, but rather strictly dependent on the MYC/PRC2-mediated activation of WNT pathway. Importantly, culturing MYC-derived mESCs in LIF/serum medium reverts them back to a LIF-dependent but PRC2/WNT-independent pluripotent state, underlying the epigenetic nature of the memory mechanism which permits their derivation. Accordingly, the expression of downstream targets of both LIF and WNT pathways (SOCS3 and FZD7) is epigenetically controlled and switched between the different pluripotent states described (Fagnocchi et al., 2016a). Solid green and red arrows indicate high or low expression on nearby genes/functions, respectively.

We recently described a MYC-driven epigenetic memory mechanism, which is able to maintain mESC identity (Fagnocchi et al., 2016a). As mentioned above, MYC-dependent mESCs rely on the activation of the WNT pathway to support their stemness, which in turn induces expression of both endogenous *myc* and *mycn* genes, establishing a positive feedback loop (Figure 2A). Importantly, mESCs maintained for long-term in MYC-dependency retain their identity, differentiation potential and pluripotency, even after deactivation of MYC-ER. On the contrary, mESCs which experienced short-term activation of MYC-ER are not able to maintain the pluripotent state upon MYC-ER deactivation and differentiate spontaneously. These evidences indicate that the MYC-driven self-reinforcing circuit, installed during MYC-dependency, is able to sustain PSCs in the absence of the instructing stimulus. In accordance, mESCs derived after MYC-ER deactivation (MYC-derived) possess a transcriptional program conserved with MYC-dependent cells and show activation of the WNT pathway, which is achieved by the endogenous MYC proteins that recruit PRC2 on WNT antagonists (Figure 3A). By definition, epigenetic regulatory mechanisms must be reversible, as they are not dictated by changes in DNA (Bonasio et al., 2010). In agreement with this, MYC-derived cells can be reprogrammed to a LIF-dependent state of pluripotency. Indeed, MYC-derived mESCs, as their parental MYC-maintained counterpart, are strictly dependent on WNT pathway activation and the PRC2 activity, while mESCs cultured in LIF/serum only depend on the JAK/STAT3 signaling (Figure 3B). By culturing MYC-derived cells in LIF/serum containing medium, they undergo a reprogramming process which render them LIF-dependent but WNT- and PRC2-independent. Of importance, this is accompanied by an epigenetic reprogramming in which WNT pathway related genes (e.g., Fzd7) switch from an active state, marked by H3K4me3 deposition on their promoter, to transcriptional repression, underlined by gaining of the H3K27me3 repressive mark. *Vice versa*, downstream effectors of the LIF/JAK/STAT3 cascade (e.g., SOCS3) re-acquire transcriptional activation, after having been repressed in both MYC-dependent and MYC-derived cells (Figure 3B). Altogether, these evidences demonstrate that MYC is able to induce a reversible epigenetic memory, which is sufficient to sustain PSCs.

## Conclusions and implications

Since MYC was first implicated in maintaining mESCs more than 10 years ago (Cartwright et al., 2005), its central role in integrating complex regulatory networks in PSCs has been tremendously reinforced and deepened. Its ability to bind specific targets and to possibly invade less specific genomic regions (Lin et al., 2012; Nie et al., 2012; Sabo et al., 2014; Walz et al., 2014), together with its *plethora* of interactions with other TFs and chromatin players (Smith et al., 2011; Tu et al., 2015), renders MYC a central hub involved in many aspects of the PSC regulation. For the same reason, the therapeutic implications of unraveling the MYC-mediated molecular mechanisms, which induce and maintain the pluripotent state, are self-evident.

Immediately after their first derivation (Takahashi et al., 2007), human iPSCs were generated from patients affected from a variety of pathologies (Dimos et al., 2008; Park et al., 2008). These patients-derived iPSCs hold the potential to generate disease models, which overcome the limits imposed by using other mammal models, such as the differences at the genomic level, in embryonic development and organ functions. Both human ESCs, either carrying spontaneous chromosomal aberrations or genetically engineered, and iPSCs from patients are now used as disease models, whose applications range from identification of the pathological molecular mechanisms, to drug discovery and toxicity testing (Zeltner and Studer, 2015; Avior et al., 2016). Furthermore, the use of patient-specific PSCs holds promises to move medical research and therapies toward more personalized approaches. Accordingly, hESCs/iPSCs may be eventually used in autologous cell replacement therapies and PSC-derivatives are now being evaluated in ongoing clinical trials for their safety and therapeutic benefits (Trounson and Dewitt, 2016). Finally, iPSCs can also be used as models for tumorigenesis (Kim and Zaret, 2015; Laplane et al., 2015). Indeed, human cells from a variety of cancers have been reprogrammed to iPSCs, in which the pluripotency state can partially suppress cancer features, by restoring the differentiation potential while reducing tumorigenicity. Nonetheless, these cancer-derived iPSCs can re-acquire the aggressive phenotype, once differentiated toward the tissue of origin, therefore representing a powerful tool to study early steps of oncogenesis, its progression, the markers and pathways involved and the response to therapeutics (Kim and Zaret, 2015; Laplane et al., 2015). In this regard, the role of MYC in controlling the regulatory networks of PSCs is particularly important as it is found up-regulated in up to the 70% of all human malignancies and, as commented in the previous sections, some of its regulated functions are shared between cancer and PSC, opening the possibility for therapeutic targeting (Dang, 2012; Ciriello et al., 2013; Gabay et al., 2014).

While the feasibility of generating PSC-based models and deriving cells for regenerative medicine have been often reported, much additional work will be required to evaluate the impact of those applications in treating multiple diseases.

## Author contributions

LF and AZ discussed the subject and wrote the manuscript.

### Conflict of interest statement

The authors declare that the research was conducted in the absence of any commercial or financial relationships that could be construed as a potential conflict of interest.

## Abbreviations

2i: two inhibitor
α-KG: α-ketoglutarate
bHLH-LZ: basic helix–loop–helix leucine-zipper
BMP4: bone morphogenetic protein 4
CDKs: cyclin-dependent kinases
CHIR: CHIR99021
CoA: acetyl-coenzyme A
dKO: MYC/MYCN double knock-out
DNMTs: DNA methyltransferases
DUSP2/7/9: dual specific phosphatases 2/7/9
EpiSCs: epiblast-derived stem cells
ESCC: ESC-specific cell-cycle regulating miRNAs
ESCs: embryonic stem cells
ETC: electron transport chain
FAD: flavin adenine dinucleotide
FGF2: basic fibroblast growth factor
H3K27me3: histone 3 lysine 27 tri-methylation
H3K4me1/2/3: histone 3 lysine 4 mono-/di-/tri-methylation
H3K9ac: histone 3 lysine 9 acetylation
H3K9me1/2/3: histone 3 lysine 9 mono-/di-/tri-methylation
HDACs: histone deacetylases
HDMs: histone demethylases
hESCs: human ESCs
ICM: inner cell mass
iPSCs: induced pluripotent stem cells
JAK-STAT3: Janus kinase–signal transducer and acti–vator of transcription 3
lncRNAs: long non-coding RNAs
MBI-IV: MYC boxes I-IV
mESCs: mouse ESCs
miRNAs: micro RNAs
MYC-ER: MYC^T58A^-estrogen receptor
NAD: adenine dinucleotide nicotinamide
ncRNAs: non-coding RNAs
NLS: nuclear localization sequence
OHT: 4-hydroxytamoxifen
OSK: OCT4/SOX2/KLF4
OSKM: OCT4/SOX2/KLF4/MYC
OSN: OCT4/SOX2/NANOG
Oxphos: oxidative phosphorylation
PcG: polycomb group proteins
PD03: PD0325901
PRC1: polycomb repressive complex 1
PRC2: polycomb repressive complex 2
PRMT7: protein arginine methyltransferase 7
PSCs: pluripotent stem cells
RB: retinoblastoma tumor suppressor
SAM: S-adenosylmethionine
TAD: transactivation domain
TFs: transcription factors
TGFβ: transforming growth factor-β
TRN: transcriptional regulatory network
TSA: trichostatin A
VPA: valproic acid.
